# Pentaradial eukaryote suggests expansion of suspension feeding in White Sea-aged Ediacaran communities

**DOI:** 10.1038/s41598-021-83452-1

**Published:** 2021-02-18

**Authors:** Kelsie Cracknell, Diego C. García-Bellido, James G. Gehling, Martin J. Ankor, Simon A. F. Darroch, Imran A. Rahman

**Affiliations:** 1 School of Earth Sciences, University of Bristol, Wills Memorial Building, Queens Road, Bristol, BS8 1RJ UK; 2 School of Biological Sciences, University of Adelaide, North Terrace Campus, Adelaide, South Australia 5005 Australia; 3 South Australian Museum, Adelaide, South Australia 5000 Australia; 4 Department of Earth Sciences and Sprigg Geobiology Centre, University of Adelaide, North Terrace Campus, Adelaide, South Australia 5005 Australia; 5 Department of Earth and Environmental Sciences, Vanderbilt University, Nashville, TN 37235-1805 USA; 6 Senckenberg Museum of Natural History, 60325 Frankfurt, Germany; 7 Oxford University Museum of Natural History, Oxford, OX1 3PW UK

**Keywords:** Palaeontology, Biomechanics

## Abstract

Suspension feeding is a key ecological strategy in modern oceans that provides a link between pelagic and benthic systems. Establishing when suspension feeding first became widespread is thus a crucial research area in ecology and evolution, with implications for understanding the origins of the modern marine biosphere. Here, we use three-dimensional modelling and computational fluid dynamics to establish the feeding mode of the enigmatic Ediacaran pentaradial eukaryote *Arkarua*. Through comparisons with two Cambrian echinoderms, *Cambraster* and *Stromatocystites*, we show that flow patterns around *Arkarua* strongly support its interpretation as a passive suspension feeder. *Arkarua* is added to the growing number of Ediacaran benthic suspension feeders, suggesting that the energy link between pelagic and benthic ecosystems was likely expanding in the White Sea assemblage (~ 558–550 Ma). The advent of widespread suspension feeding could therefore have played an important role in the subsequent waves of ecological innovation and escalation that culminated with the Cambrian explosion.

## Introduction

The late Ediacaran (~ 571–541 Ma) was a pivotal interval in Earth’s history, which saw the initial radiation of large and complex multicellular eukaryotes (the so-called ‘Ediacaran macrobiota’), including some of the first animals^1–3^. Although Ediacaran ecosystems were, for many years, thought to have been fundamentally different from Cambrian ones^4,5^, there is growing evidence that they were more similar than previously thought, especially in terms of the construction and organization of communities, presence of key feeding strategies, and diversity of life modes^6–9^. One of the most important ecological innovations that emerged in the Ediacaran, and which is thought to have played a crucial role in structuring Phanerozoic communities, is macroscopic suspension feeding. Benthic suspension feeders are responsible for removing suspended organic particles from the water column, thereby reducing primary production and increasing the retention time for suspended particles on the seafloor. This provides a link between the pelagic and benthic realms and exerts a powerful control over rates and patterns of energy transport^10–12^. The evolution of benthic suspension feeding therefore marked a permanent step-increase in nutrient and energy fluxes to the sediment–water interface, and may have represented a primary ecological driver for the Cambrian explosion^13^.

Establishing when suspension feeding by macroscopic organisms became dominant in ecosystems is a key goal in evolutionary biology and geobiology. The appearance of the probable suspension feeders *Cloudina* and *Namacalathus* as major components of reefs in the latest Ediacaran ‘Nama’ assemblage (~ 550–541 Ma) indicates that suspension feeding had become an important and widespread ecological strategy shortly before the onset of the Cambrian^12,14^. Analysis of older Ediacaran communities (‘White Sea’ assemblage, ~ 558–550 Ma) suggests that competition for food within the water column became a significant factor at this time^15^, but the aberrant morphologies of many taxa from this assemblage, which lack analogues among modern species, makes determining their feeding modes problematic. Rahman et al.^6^ suggested that the triradial taxon *Tribrachidium* was a benthic suspension feeder, but White Sea-aged communities encompass a wide diversity of taxa which have yet to be analyzed in this context. It is therefore uncertain if suspension feeding played a prominent role in structuring benthic communities prior to 550 million years ago.

The White Sea assemblage, represented by fossils from the White Sea Region of Russia and the Flinders Ranges of South Australia, marks the apex of Ediacaran diversity^16^. *Arkarua adami* is one of the most enigmatic fossils from this assemblage, characterized by a small, disc-shaped body with five grooves radiating from a central depression on the upper surface. It is thought to have been sessile, resting on the seafloor in life^17^. Owing to its pentaradial body plan, *Arkarua* has been interpreted as the earliest known echinoderm^17,18^, but this phylogenetic position is debated^19,20^. Laflamme et al.^21^ suggested that most White Sea taxa, including *Arkarua*, were osmotrophs, feeding by absorbing dissolved organic carbon, as has been proposed for a wide range of Ediacaran organisms^21,22^. However, an alternative possibility, indicated by the general morphological similarity with the Cambrian edrioasteroid echinoderms *Cambraster* and *Stromatocystites*^17^, is that *Arkarua* was a suspension feeder, passively feeding on particles suspended in water, as inferred for edrioasteroids^23,24^. Here, we use a virtual modelling approach called computational fluid dynamics (CFD) to visualize water flow around putative feeding structures for models of *Arkarua*, and compare the resulting flow patterns to those produced by models of *Cambraster* and *Stromatocystites*. Using these data, we test the hypothesis that *Arkarua* was a benthic suspension feeder. The results allow us to build a more complete picture of White Sea assemblage palaeoecology, shedding light on the importance of suspension feeding in the Ediacaran.

## Material and methods

### Fossil specimens

**Figure 1:**
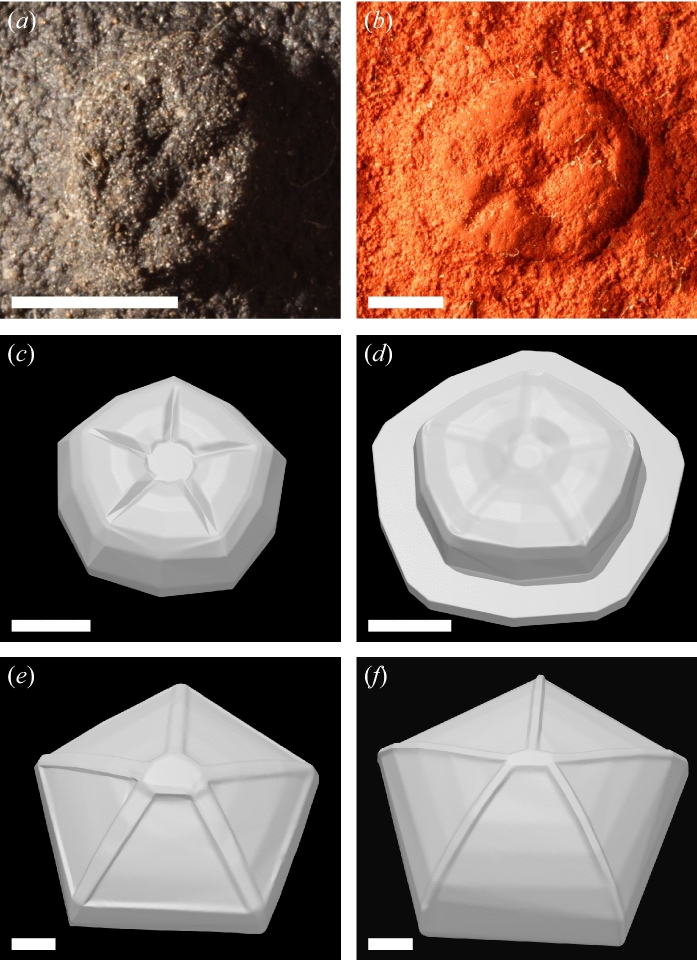
(**a**) Cast of *Arkarua adami* (SAMP P 40310) from Devil's Peak, Flinders Ranges, South Australia. (**b**) Cast of *Arkarua adami* (SAM P 26768) from the Chace Range, Flinders Ranges, South Australia. (**c**) Three-dimensional digital model of *Arkarua* morphotype 1. (**d**) Three-dimensional digital model of *Arkarua* morphotype 2. (**e**) Three-dimensional digital model of *Cambraster*. (**f**) Three-dimensional digital model of *Stromatocystites*. Scale bars = 2 mm.

**Figure 2:**
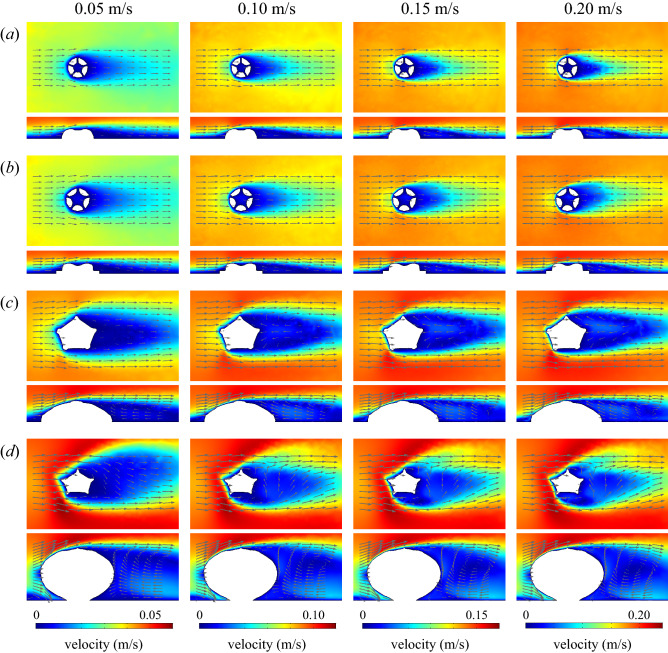
Two-dimensional surface plots (horizontal and vertical cross-sections) of velocity magnitude with flow vectors (size of arrows proportional to natural logarithm of flow velocity magnitude) at four different inlet velocities (0.05–0.20 m/s). (**a**) *Arkarua* morphotype 1. (**b**) *Arkarua* morphotype 2. (**c**) *Cambraster*. (**d**) *Stromatocystites*. Direction of ambient flow from left to right.

*Arkarua adami* comes from the Nilpena Member of the Rawnsley Quartzite (~ 555 Ma) in the Flinders Ranges, South Australia^25^. Fossils are preserved as external moulds in fine- to medium-grained sandstones, with some beds showing evidence of unidirectional current ripples and micro-scour. Deposition is thought to have occurred at storm wave base on an open marine shelf^17,26,27^. Two morphotypes have been described: (1) smaller (~ 4–5 mm in diameter) hemispherical forms (Fig. 1a) and (2) larger (~ 6–10 mm in diameter) discoidal forms with a marginal rim (Fig. 1b). Measurements of specimens in the collections of the South Australian Museum (SAM) are provided in Supplementary Table S1.

### Digital modelling

Three-dimensional digital models of the two *Arkarua* morphotypes (Fig. 1c,d) and the Cambrian edrioasteroids *Cambraster cannati* (Fig. 1e) and *Stromatocystites pentangularis* (Fig. 1f) were created using box modelling^28^ in Blender v. 2.79 (www.blender.org). Photographs of fossil specimens and published reconstructions^17,29,30^ were used as background images. In addition, virtual reconstructions of *Arkarua* specimens, generated with photogrammetry (Supplementary Information), were used as references to guide box modelling of the *Arkarua* morphotypes. For each model, a cube was created and then subdivided using loop cuts to increase the number of elements. Edges and vertices of the cube were translated, rotated and/or scaled to match the outlines of the reference images/reconstructions in different views. To represent more complex parts, additional elements were created by extruding faces of this object. Models were then scaled to life size, with model diameters (5.8 mm for *Arkarua* morphotype 1, 7.5 mm for *Arkarua* morphotype 2, 13 mm for *Cambraster* and 14 mm for *Stromatocystites*) obtained from measurements of fossil specimens, whereas model heights (1.65 mm for *Arkarua* morphotype 1, 1.8 mm for *Arkarua* morphotype 2, 3.6 mm for *Cambraster* and 9 mm for *Stromatocystites*) were estimated based on the relative dimensions of published reconstructions^17,29,30^. Models were exported from Blender and converted into non-uniform rational basis spline surfaces in Geomagic Studio 2012 (www.geomagic.com). To evaluate the feasibility of an osmotrophic feeding mode, we calculated surface area-to-volume (SA:V) ratios for each of the four models and compared them with extant and extinct osmotrophs. Digital models are available from Zenodo: 10.5281/zenodo.4497656.

### Computational fluid dynamics

CFD simulations were carried out in COMSOL Multiphysics v. 5.4 (www.comsol.com) following established protocols^6,9^. A three-dimensional half cylinder, measuring 182 mm in length and 124 mm in diameter, was used as the computational domain (Supplementary Fig. S1a). Models were fixed to the lower boundary of this domain, which extended at least three times the length of the model upstream, ten times the length of the model downstream and five times the size of the model in all other directions. The physical properties of water (density = 1000 kg/m^3^, dynamic viscosity = 0.001 Pa·s) were assigned to the space surrounding the model, with a velocity inlet defined at the upstream end of the domain and a zero-pressure outlet at the downstream end. A no-slip boundary condition was assigned to the lower surface of the domain and the surfaces of the model, with a slip boundary condition assigned to the top and sides of the domain. The domain was meshed using free tetrahedral elements, with thin layers of prismatic elements inserted at the fluid–solid interface (Supplementary Fig. S1b,c). The shear-stress transport turbulence model was used to solve the Reynolds-averaged Navier–Stokes equations, with a stationary solver used to compute the steady-state solution. A sensitivity analysis was carried out to determine the optimal mesh size for each model (Supplementary Information; Supplementary Tables S2–S5), which was then used in all subsequent simulations.

A total of four inlet velocities ranging from 0.05 to 0.20 m/s (Reynolds numbers of 285–2580; model diameter taken as the characteristic dimension) were simulated for each model, reflecting ambient current velocities in modern relatively deeper-water environments^31,32^ analogous to those inhabited by *Arkarua*. This velocity range is supported by sedimentological evidence including the grain size and the presence of bedforms such as current ripples and micro-scour^17,26,27^, which indicate flow velocities were regularly greater than 0.10 m/s^33^. The same inlet velocities were simulated for *Cambraster* and *Stromatocystites*, which are thought to have inhabited relatively high-energy and low-energy (respectively) offshore environments^30^.

Simulations were performed with models at three different orientations to the inlet (0°, 36° and 324°). In addition, simulations were repeated for both *Arkarua* morphotypes (with models orientated at 0° to the inlet) with model heights increased by 15% and 30% to account for possible diagenetic compaction of the sediment and ensuing compression of *Arkarua* specimens^17^, which might have caused us to underestimate the original relief of the living organisms. To test between osmotrophy and suspension feeding, we visualized CFD results as two-dimensional and three-dimensional plots showing flow patterns around the models. CFD results files are available from Zenodo: 10.5281/zenodo.4497656.

## Results

### SA:V ratios

Surface area-to-volume (SA:V) ratios calculated for the four digitally-modelled organisms (Fig. 1c–f) were within a narrow range of 0.61–2.48 mm^–1^. The two *Arkarua* models had slightly higher values (2.10 and 2.48 mm^–1^) than *Cambraster* and *Stromatocystites* (1.22 and 0.61 mm^–1^, respectively) (Table 1).

**Table 1:** Surface area, volume and surface area-to-volume ratios for digital models of *Arkarua*, *Cambraster* and *Stromatocystites*.

Model	Surface area (mm^2^)	Volume (mm^3^)	Surface area-to-volume ratio (mm^−1^)
*Arkarua* morphotype 1	65.89	31.45	2.10
*Arkarua* morphotype 2	110.12	44.46	2.48
*Cambraster*	272.96	223.40	1.22
*Stromatocystites*	390.04	642.25	0.61

### Computational fluid dynamics

In all the CFD simulations, the velocity of fluid flow decreased as it approached the digital model and the lower boundary of the domain, with a velocity gradient (the boundary layer) developed in the immediate vicinity of these surfaces (Fig. 2; Supplementary Figs. S2, S4, S6, S8, S10, S12). The thickness of the boundary layer decreased as the inlet velocity increased, consistent with theoretical expectations. A region of recirculating flow (the wake) was seen downstream of the models (Fig. 2; Supplementary Figs. S2, S4, S6, S8, S10, S12). The size of the wake increased with the size of the model.

**Figure 3:**
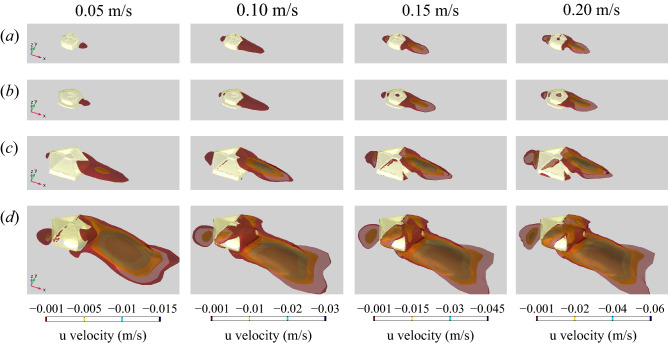
Three-dimensional isosurface plots of negative values of velocity component u (parallel to the x-axis) at three different inlet velocities (0.05–0.20 m/s). (**a**) *Arkarua* morphotype 1. (**b**) *Arkarua* morphotype 2. (**c**) *Cambraster*. (**d**) *Stromatocystites*. Direction of ambient flow from top left to bottom right.

In the *Arkarua* models, an isolated pocket of low velocity flow was observed within the central depression and associated grooves at all modelled orientations and heights (Fig. 2a,b; Supplementary Figs. S2, S4, S10, S12). Additionally, in the original models of *Arkarua* morphotypes 1 and 2, localized areas of reversed flow, represented by negative values of velocity component u (parallel to the flow direction), occurred in the central depression when the inlet velocity was greater than or equal to 0.20 or 0.15 m/s, respectively (Fig. 3a,b; Supplementary Figs. S3, S5). For the *Arkarua* models with increased heights, reversed flow within the central depression also occurred at slightly lower velocities (0.15 m/s or greater for morphotype 1 and 0.10 m/s or greater for morphotype 2) (Supplementary Figs. S11, S13). In the edrioasteroid models, low velocity flow over the mouth and downstream ambulacra was seen regardless of orientation (Fig. 2c,d; Supplementary Figs. S6, S8), with areas of reversed flow adjacent to the downstream ambulacra at inlet velocities of 0.10 m/s or greater (Fig. 3c,d; Supplementary Figs. S7, S9).

## Discussion

The calculated SA:V ratios (Table 1) argue against osmotrophy as the primary feeding mode for *Arkarua*. Osmotrophy relies on a high SA:V ratio to enhance the uptake of dissolved organic matter through osmosis^22,34^, but the values obtained for models of *Arkarua* (2.10 and 2.48 mm^–1^), *Cambraster* (1.22 mm^–1^) and *Stromatocystites* (0.61 mm^–1^) are much lower than extant osmotrophic megabacteria, which range from 8–20,000 mm^–1^ (see Laflamme et al.^22^). Ediacaran organisms interpreted as osmotrophs also had generally higher SA:V ratios than *Arkarua*; for example, theoretical models of the rangeomorph *Fractofusus* with fractally-branching modules have SA:V ratios of 2–10,000 mm^–1^ (see Laflamme et al.^22^). Moreover, *Arkarua* lacks any of the morphological adaptations for enhancing the SA:V ratio seen in rangeomorphs, such as constructional flattening or fractal branching^21,22^.

The results of our CFD simulations are also incompatible with an osmotrophic feeding mode for *Arkarua*, and instead strongly suggest that it was a suspension feeder. If *Arkarua* was an osmotroph, we would expect to see fluid flow evenly distributed across all surfaces of the models, maximizing the opportunities for uptake of dissolved organic carbon^35^. However, our results reveal that low velocity flow and, at higher inlet velocities, reversed flow was concentrated over specific parts of the models, i.e. the central depression and associated grooves (Figs. 2a,b, 3a,b; Supplementary Figs. S2–S5, S10–S13). Such flow patterns are more consistent with passive feeding on particles suspended in water using specialized feeding structures^36^, as suggested for some other Ediacaran organisms based on computer simulations of fluid flows^6,9,37^. Furthermore, our CFD simulations for the edrioasteroids *Cambraster* and *Stromatocystites* (which are widely regarded as passive suspension feeders^23,24^) reveal very similar flow patterns, with pockets of low velocity and reversed flow adjacent to the central mouth and downstream ambulacra (Figs. 2c,d, 3c,d; Supplementary Figs. S6–S9), further strengthening our inference of suspension feeding in *Arkarua*. While it is possible that osmotrophy could have served as a supplemental food source, as in some extant marine animals^38–40^, it would not have been an effective means of gathering nutrients in *Arkarua*.

Concentration of low velocity flow over the central depression and associated grooves in *Arkarua* indicates these structures were important for feeding, as previously suggested^17^. The observed flow patterns are most consistent with particle capture through gravitational deposition, with particles denser than water falling out of suspension over feeding structures under the influence of gravity^41,42^. This feeding strategy has been reported in a variety of modern sessile marine invertebrates, including bivalves, corals and crinoids^43–46^. Flow may have been channelled along the grooves towards the central depression, which presumably served as an opening into the body cavity where nutrients were absorbed.

Reconstruction of *Arkarua* as a benthic suspension feeder illustrates that the diversity and abundance of suspension feeding taxa in the White Sea assemblage was greater than previously thought. CFD analyses have shown that the White Sea taxon *Tribrachidium* was also a suspension feeder^6^, and hence the low-relief, hemispherical morphologies exhibited by both *Arkarua* and *Tribrachidium* probably evolved to enhance capture of suspended food particles just above the sediment–water interface. Moreover, the triradial body plans of other Ediacaran taxa, such as *Albumares*, *Anfesta*, *Hallidaya*, *Rugoconites* and *Skinnera*^47^, suggest that these too would have interacted with moving currents in a similar fashion, and thus likely also functioned as suspension feeders. White Sea ecosystems were therefore comprised of a substantial diversity and overall proportion of suspension-feeding organisms, which lived alongside phototrophs^48^, mobile mat grazers^49^, saprotrophs^50^, detritivores^7^ and osmotrophs^21^ in surprisingly complex benthic communities. This scenario invites two crucial questions with broad relevance for reconstructing ecological and evolutionary dynamics during the Neoproterozoic rise of animals. Firstly, why did so many (apparently unrelated) suspension feeders adopt a low-relief and hemispherical body plan? Secondly, what effects might the proliferation of suspension feeders have had on the structure and function of Ediacaran communities?

The morphology of extant sessile suspension feeders reflects, in part, a trade-off between feeding and stability. Adaptations that increase the height of an organism above the sediment–water interface allow it to take advantage of higher current velocities for feeding, but will also increase drag and, hence, the chances of dislodgement^36,51^. In this context, a low-relief, hemispherical body plan may represent one possible locally optimal morphology, minimizing drag and thus enhancing stability on the seafloor, while at the same time creating patterns of fluid flow that would have enabled passive suspension feeding^6^. A hemispherical aspect reduces drag and enables feeding in all orientations to current equally, and therefore might represent an adaptation to life in environments characterized by shifting current directions^6,37^. Hemispherical body plans have been adopted by sessile suspension feeders in a wide variety of metazoan phyla, including cnidarians, echinoderms, arthropods (e.g. barnacles) and chordates (e.g. *Cnemidocarpa* tunicates)^36,51^. Consequently, we suggest that the abundance of hemispherical body plans among Ediacaran macrobiota in the White Sea assemblage reflects both the expansion of benthic ecosystems from deep into shallow water environments characterized by strong and variable currents^52^ and the increasingly widespread availability of suspended food at low heights in the water column. With seafloor microbial mats being extensive at this time, turbulent flow near the sediment–water interface would have resulted in a large volume of re-suspended organic matter and bacteria. Moreover, recent biomarker work performed on White Sea-aged sediments suggests that algae was an important food source in shallow-water environments^53^, and thus could have been a valuable resource for benthic suspension-feeding communities.

The earliest benthic suspension feeders probably appeared in the ‘Avalon’ assemblage (~ 571–560 Ma). Although the vast majority of Avalon-aged taxa are generally regarded as osmotrophs^21,22^, the enigmatic triangular-shaped fossil *Thectardis* has been interpreted as a sponge on the basis of its aspect ratio^54^ (although see^55^), and could thus represent the earliest link between pelagic and benthic realms. Our results suggest this feeding behaviour had become more widespread by the White Sea assemblage, appearing in at least three putative clades of Ediacaran macrobiota (erniettomorphs, pentaradialomorphs and triradialomorphs^6,9^), in addition to possible sponges like *Coronacollina* and *Paleophragmodictya*^56–58^. As well as the expansion of suspension feeding strategies into new taxonomic groups, the White Sea assemblage was also characterized by increased ecological tiering, with an abundance of low-tiered suspension feeders just above the sediment–water interface. This trend extended into the latest Ediacaran Nama assemblage, with the expansion of likely suspension feeders into higher tiers (e.g. *Corumbella*^59^) and new ecological niches, such as reef crests (e.g. *Cloudina* and *Namacalathus*^12,14^). Consequently, our reconstruction of *Arkarua* as a benthic suspension feeder potentially highlights a late Ediacaran rise to prominence of suspension feeding in benthic ecosystems.

The organic matter captured by suspension feeders is typically either converted into biomass or excreted to the sediment, where it forms an invaluable energy source for organisms that rarely (or never) venture up higher into the water column^60^. The putative late Ediacaran increase in energy transport from the water column to the sediment surface would thus have represented a permanent step-increase in the bioavailable carbon at the sediment–water interface, and could have fuelled several of the dramatic ecological and evolutionary innovations seen during this interval, including the appearance of mobile benthic organisms, increased diversity of body plans, and exploitation of the sediment–water interface by bilaterian tracemakers (the ‘second wave’ of Ediacaran innovation proposed by Droser and Gehling^61^). In this light, the dramatic radiation in infaunal deposit-feeding behaviours evident in the succeeding Nama assemblage^62–64^ may have been an ecological response to a step-increase in benthic food availability. We acknowledge, however, that this model is speculative given that current understanding of Ediacaran feeding modes is incomplete. For example, if rangeomorphs are interpreted as suspension feeders^65^ rather than osmotrophs^21,22^, this would render Avalon-aged communities as almost entirely composed of suspension feeders. Moreover, the feeding modes of most White Sea taxa remain poorly understood; additional studies focussed on the palaeobiology of individual taxa are needed. Nevertheless, the growing number of probable suspension feeders from White Sea-aged communities raises the possibility that the link between pelagic and benthic realms strengthened during the late Ediacaran, resulting in increased energy flux to the sediment surface, and potentially supporting a diversification in benthic life habits. We note that this hypothesis is different from, but not mutually exclusive to, other resource-based models seeking to explain the dramatic increase in taxonomic and ecological diversity across the Avalon–White Sea transition^15,66^.

In summary, we show that the enigmatic Ediacaran pentaradial organism *Arkarua adami* was a passive suspension feeder, which took advantage of flow patterns created by its radially symmetrical and approximately hemispherical body plan. *Arkarua* thus joins a growing list of probable suspension feeders in the late Ediacaran White Sea assemblage, and suggests that a key pillar of the Phanerozoic marine carbon cycle – linkage between pelagic and benthic ecosystems – may have expanded from the Avalon to White Sea assemblages, ~ 571–550 Ma. In the absence of any obvious temporal correlation between putative environmental shifts (including the oxygenation state of global oceans) and the increases in both biological and ecological complexity in the late Ediacaran^67^, we propose that the expansion of benthic suspension feeding in the White Sea assemblage played an important role in shaping the waves of innovation that began in the late Ediacaran, and culminated with the Cambrian explosion^2,3,68^.

## Supplementary Information

Supplementary Information

## Data Availability

Digital models in STL and IGES formats and CFD results files in MPH and DOCX formats are available from Zenodo: 10.5281/zenodo.4497656.
