# Supplementary material for: Pentaradial eukaryote suggests expansion of suspension feeding in White Sea-aged Ediacaran communities
Source: Sci Rep. 2021 Feb 18;11:4121. doi: 10.1038/s41598-021-83452-1 (PMC7893023; doi:10.1038/s41598-021-83452-1)
Supplement: Supplementary file 1 — Supplementary Information [file 41598_2021_83452_MOESM1_ESM.docx]

**Supplementary Information**

**Pentaradial eukaryote suggests expansion of suspension feeding in White Sea-aged Ediacaran communities**

Kelsie Cracknell^1^, Diego C. García-Bellido^2,3^, James G. Gehling^3^, Martin J. Ankor^4^, Simon A. F. Darroch^5,6^ and Imran A. Rahman^7,*^

^1^School of Earth Sciences, University of Bristol, Wills Memorial Building, Queens Road, Bristol BS8 1RJ, UK.

^2^School of Biological Sciences, University of Adelaide, North Terrace Campus, Adelaide, South Australia 5005, Australia.

^3^South Australian Museum, Adelaide, South Australia 5000, Australia.

^4^Department of Earth Sciences and Sprigg Geobiology Centre, University of Adelaide, North Terrace Campus, Adelaide, South Australia 5005, Australia.

^5^Department of Earth and Environmental Sciences, Vanderbilt University, Nashville, TN 37235-1805, USA.

^6^Senckenberg Museum of Natural History, 60325 Frankfurt, Germany.

^7^Oxford University Museum of Natural History, Oxford OX1 3PW, UK.

*email: [imran.rahman@oum.ox.ac.uk](mailto:imran.rahman@oum.ox.ac.uk)

**Photogrammetry**

Virtual reconstructions of casts of the holotype of *Arkarua adami* (SAM P 26768) and five paratypes (SAM P 26770, 26771, 26772, 40562 and 41147) were created with photogrammetry. An Olympus E-M1 Mark II camera with a 60 mm macro lens was used for photography, giving a field of view of ~17.4 x 13 mm and a resolution of ~300 pixels per mm. Casts were photographed from directly above and at oblique angles, providing a series of images with greater than 60% overlap. Photogrammetric processing was performed using Agisoft Metashape (www.agisoft.com), with the resulting meshes scaled to the correct dimensions using 3DReshaper (www.3dreshaper.com).

**Sensitivity analysis**

To establish the optimal mesh size for use in computational fluid dynamics (CFD) simulations (i.e. one that ensures computational accuracy while minimizing simulation time), a sensitivity analysis was undertaken. Here, CFD simulations were repeated for the digital models of the two *Arkarua* morphotypes, *Cambraster* and *Stromatocystites* at the same orientation to the inlet (0°) using five different mesh sizes. To compare the results, drag forces were computed by integrating the total stress parallel to the flow direction, with the dimensionless coefficients of drag (C_D_) calculated using the following formula:

$$\text{C}\text{D}\text{ = }\frac{\text{2}\text{F}\text{D}}{\text{ρU}\text{2}\text{A}}$$

where *F_D_* is the drag force exerted by the fluid (N), *ρ* is the density of the fluid (kg/m^3^), *U* is the characteristic velocity (m/s) and *A* is the characteristic area (m^2^). The projected frontal area was taken as the characteristic area.

CFD results were treated as mesh independent when the drag coefficients obtained differed by less than 3% compared to the next coarsest mesh (Tables S2–S5). Based on these criteria, we selected a mesh for each model (ranging from 372,125 to 555,026 elements in size) that was used in all subsequent analyses.

**Supplementary figures**

**
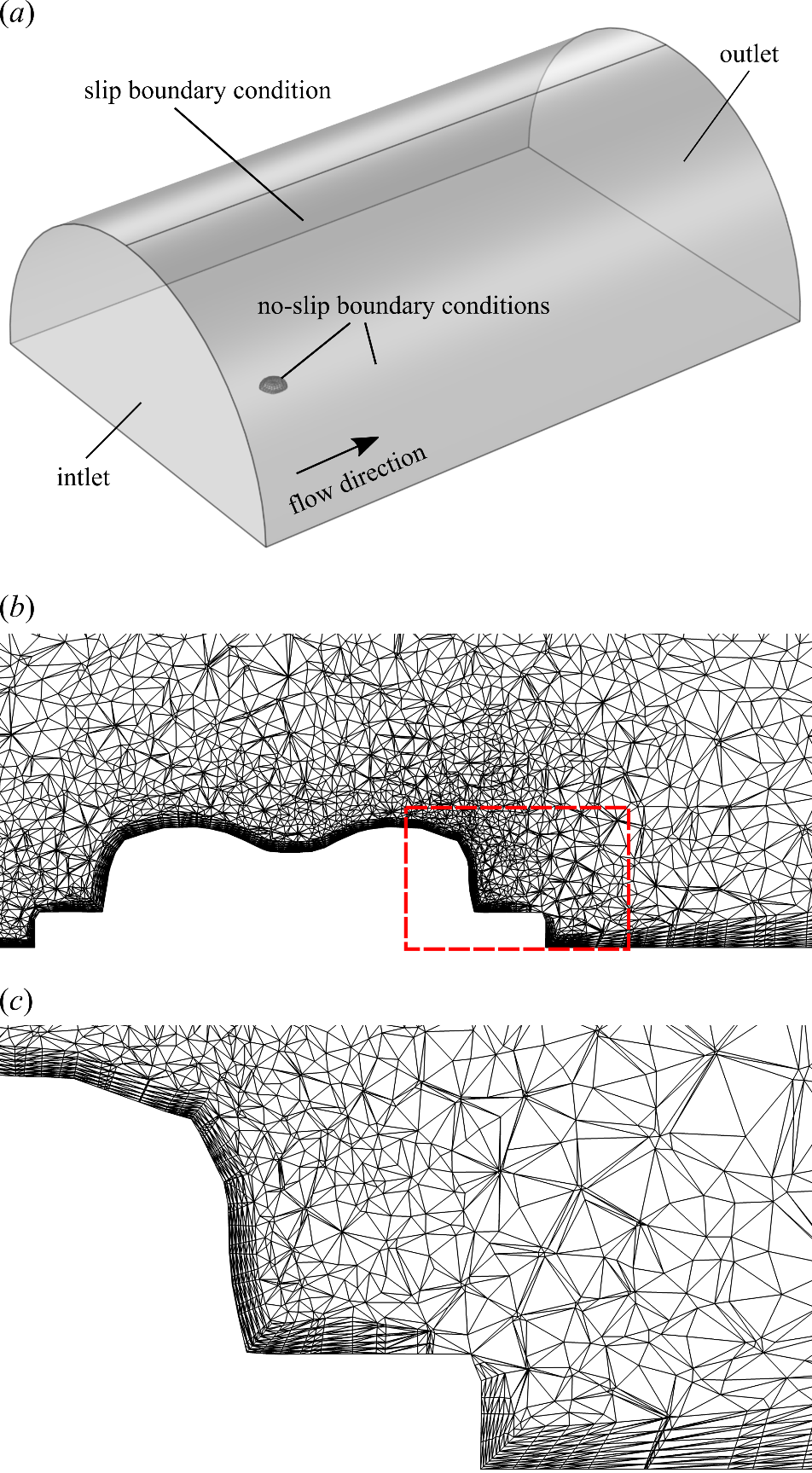
**

**Figure S1.** (*a*) Computational domain used in CFD simulations. (*b*,*c*) Vertical cross-section through mesh used in CFD simulations for *Arkarua* morphotype 2 (red dashed box in *b* marks position of *c*).


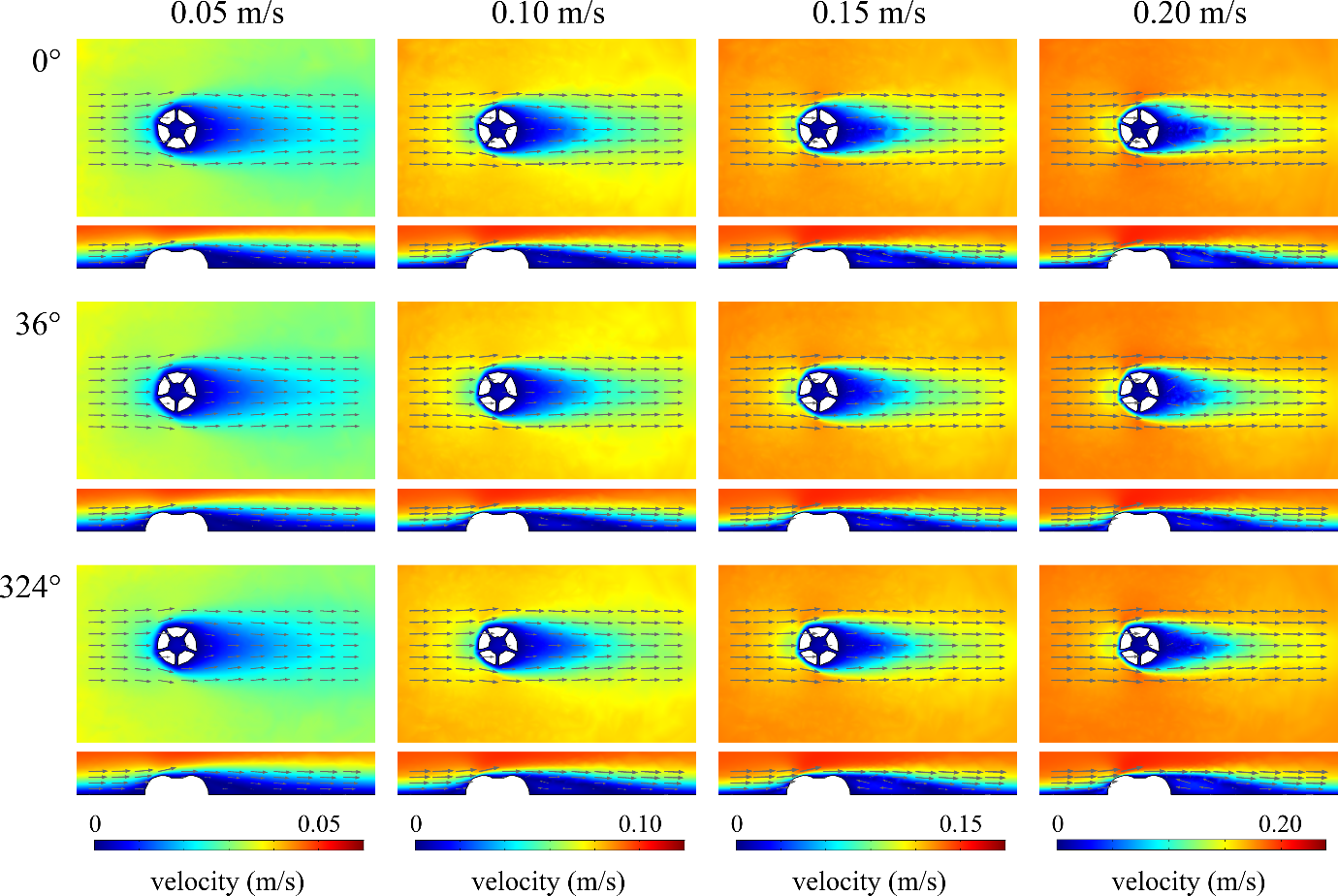


**Figure S2.** Two-dimensional surface plots (horizontal and vertical cross-sections) of velocity magnitude with flow vectors (size of arrows proportional to natural logarithm of flow velocity magnitude) at four different inlet velocities (0.05–0.20 m/s) for *Arkarua* morphotype 1 oriented at 0°, 36° and 324° to the inlet. Direction of ambient flow from left to right.


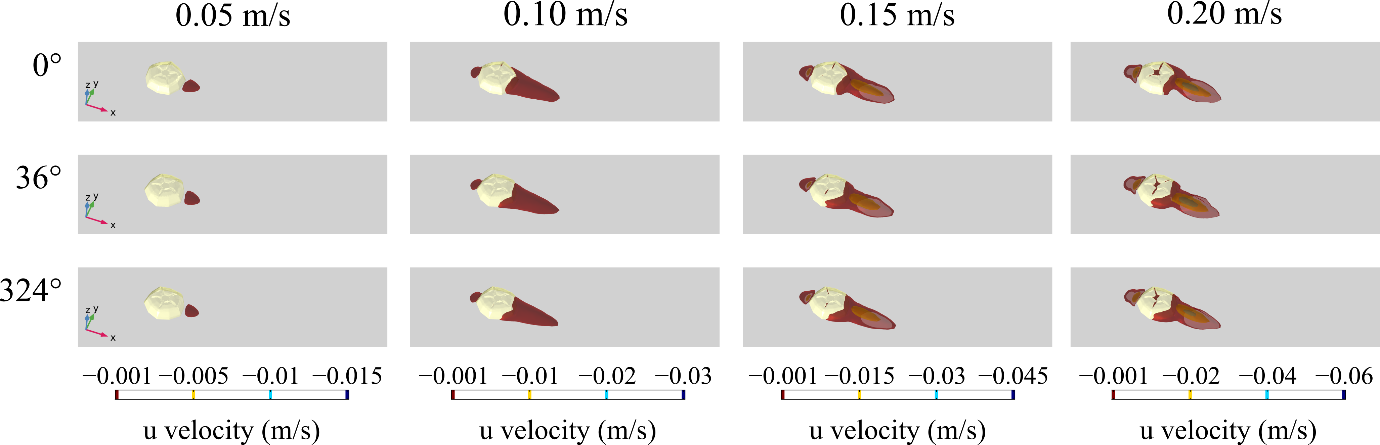


**Figure S3.** Three-dimensional isosurface plots of negative values of velocity component u (parallel to the x-axis) at four different inlet velocities (0.05–0.20 m/s) for *Arkarua* morphotype 1 oriented at 0°, 36° and 324° to the inlet. Direction of ambient flow from top left to bottom right.

**
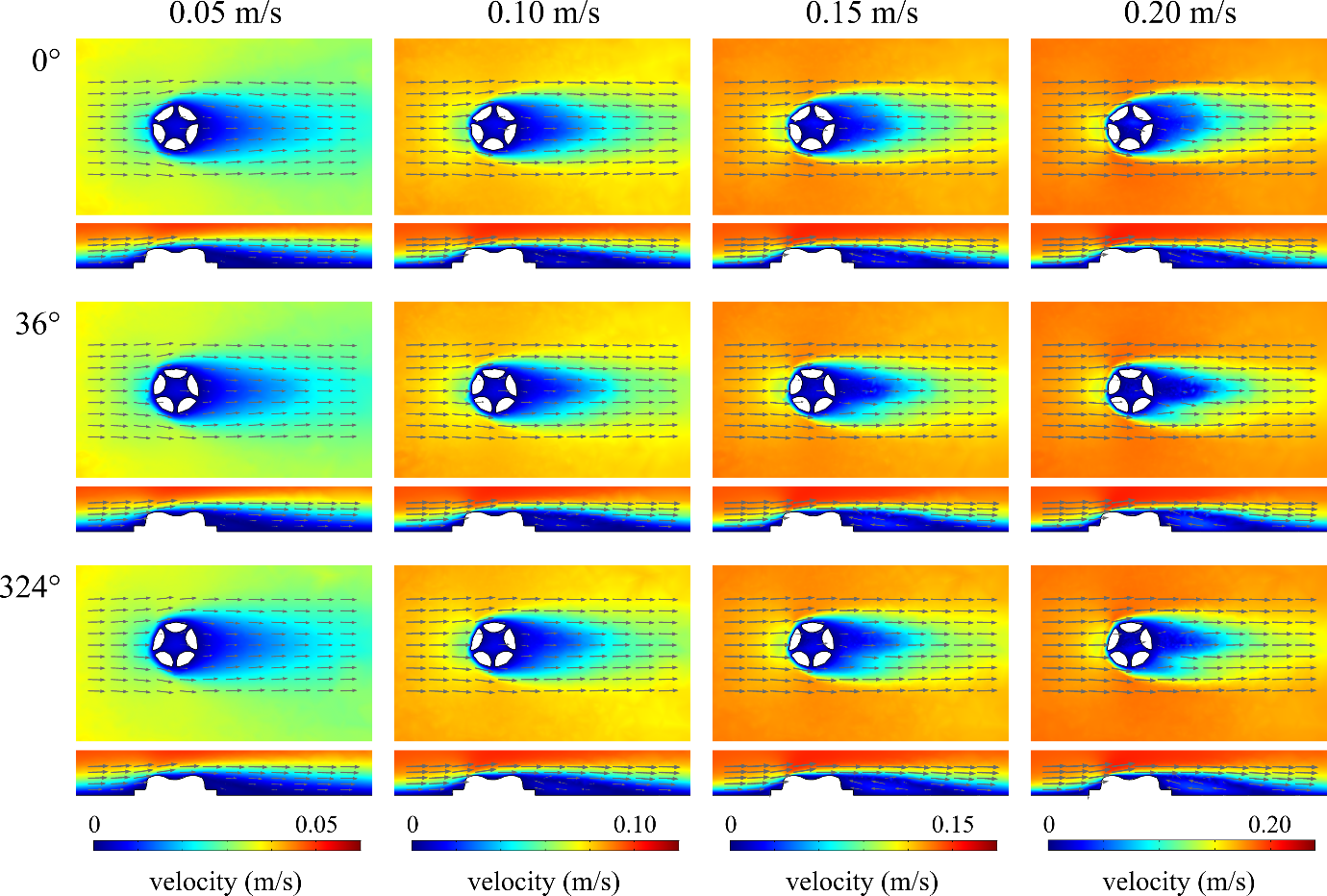
**

**Figure S4.** Two-dimensional surface plots (horizontal and vertical cross-sections) of velocity magnitude with flow vectors (size of arrows proportional to natural logarithm of flow velocity magnitude) at four different inlet velocities (0.05–0.20 m/s) for *Arkarua* morphotype 2 oriented at 0°, 36° and 324° to the inlet. Direction of ambient flow from left to right.


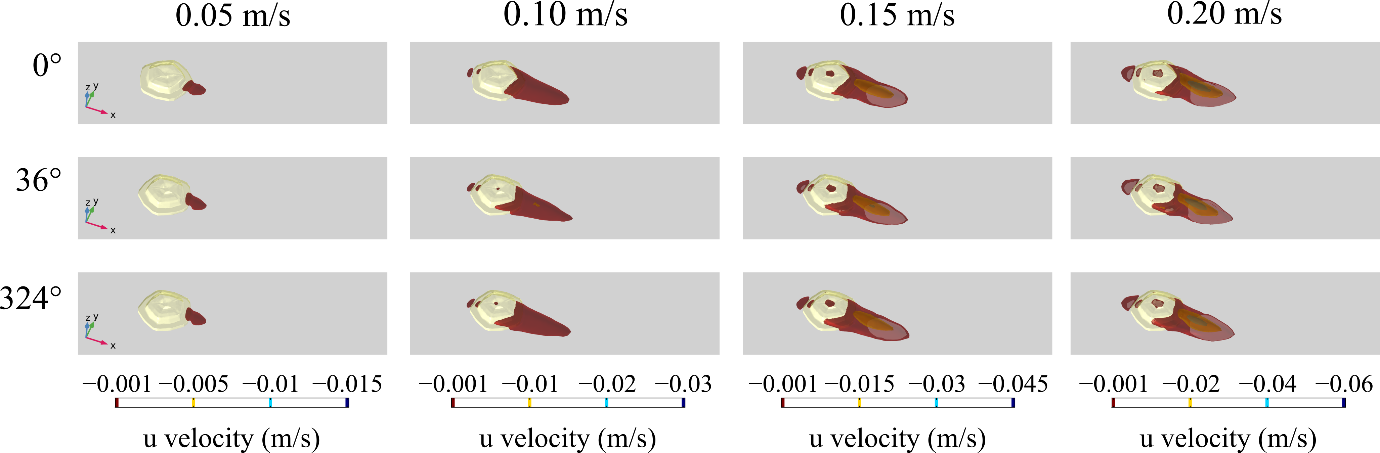


**Figure S5.** Three-dimensional isosurface plots of negative values of velocity component u (parallel to the x-axis) at four different inlet velocities (0.05–0.20 m/s) for *Arkarua* morphotype 2 oriented at 0°, 36° and 324° to the inlet. Direction of ambient flow from top left to bottom right.

**
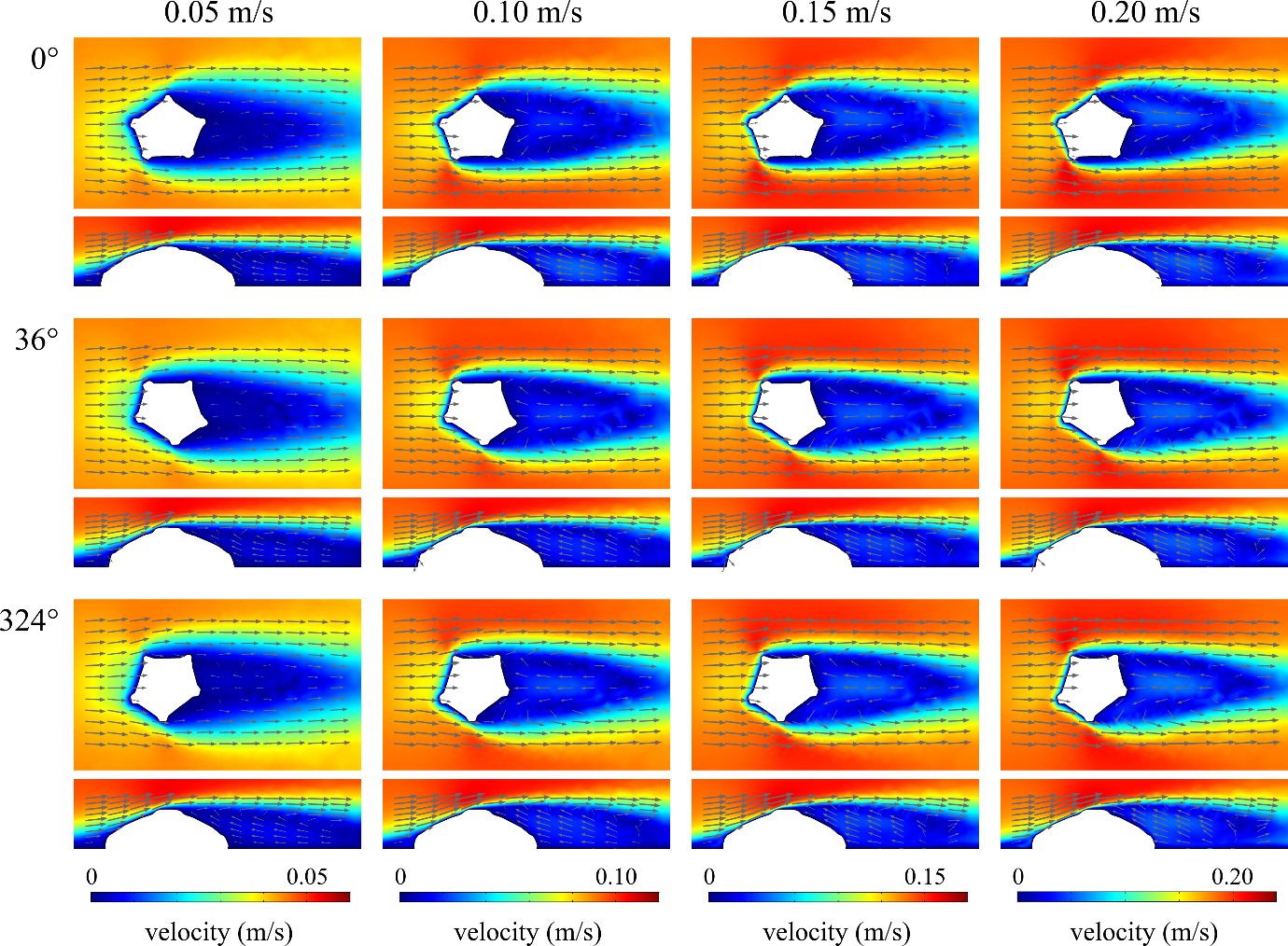
**

**Figure S6.** Two-dimensional surface plots (horizontal and vertical cross-sections) of velocity magnitude with flow vectors (size of arrows proportional to natural logarithm of flow velocity magnitude) at four different inlet velocities (0.05–0.20 m/s) for *Cambraster* oriented at 0°, 36° and 324° to the inlet. Direction of ambient flow from left to right.


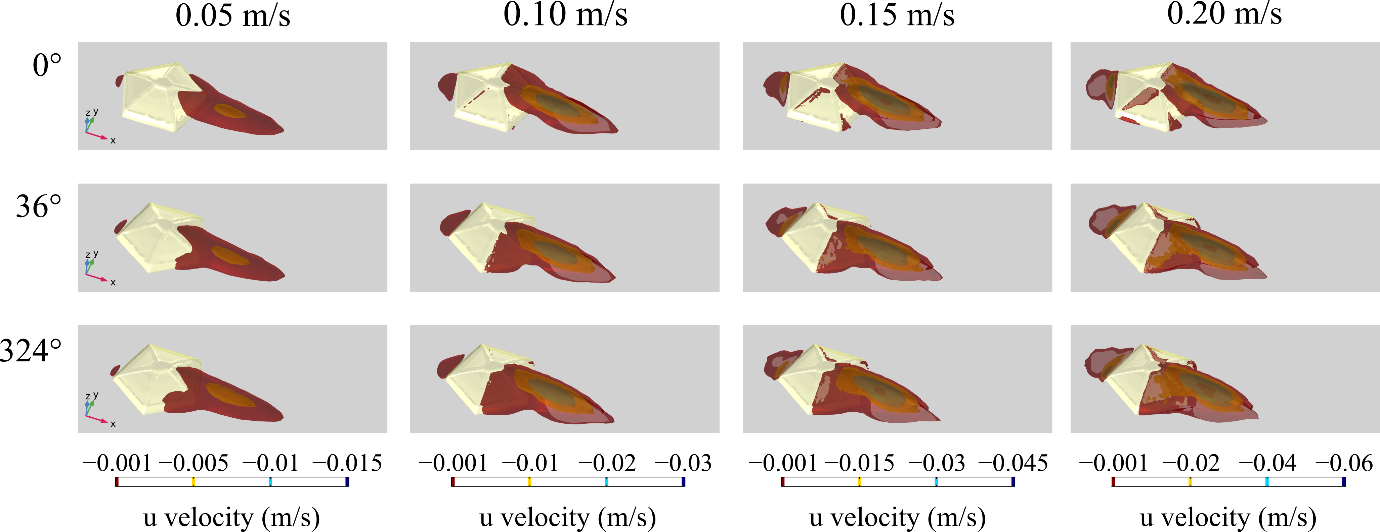


**Figure S7.** Three-dimensional isosurface plots of negative values of velocity component u (parallel to the x-axis) at four different inlet velocities (0.05–0.20 m/s) for *Cambraster* oriented at 0°, 36° and 324° to the inlet. Direction of ambient flow from top left to bottom right.

**
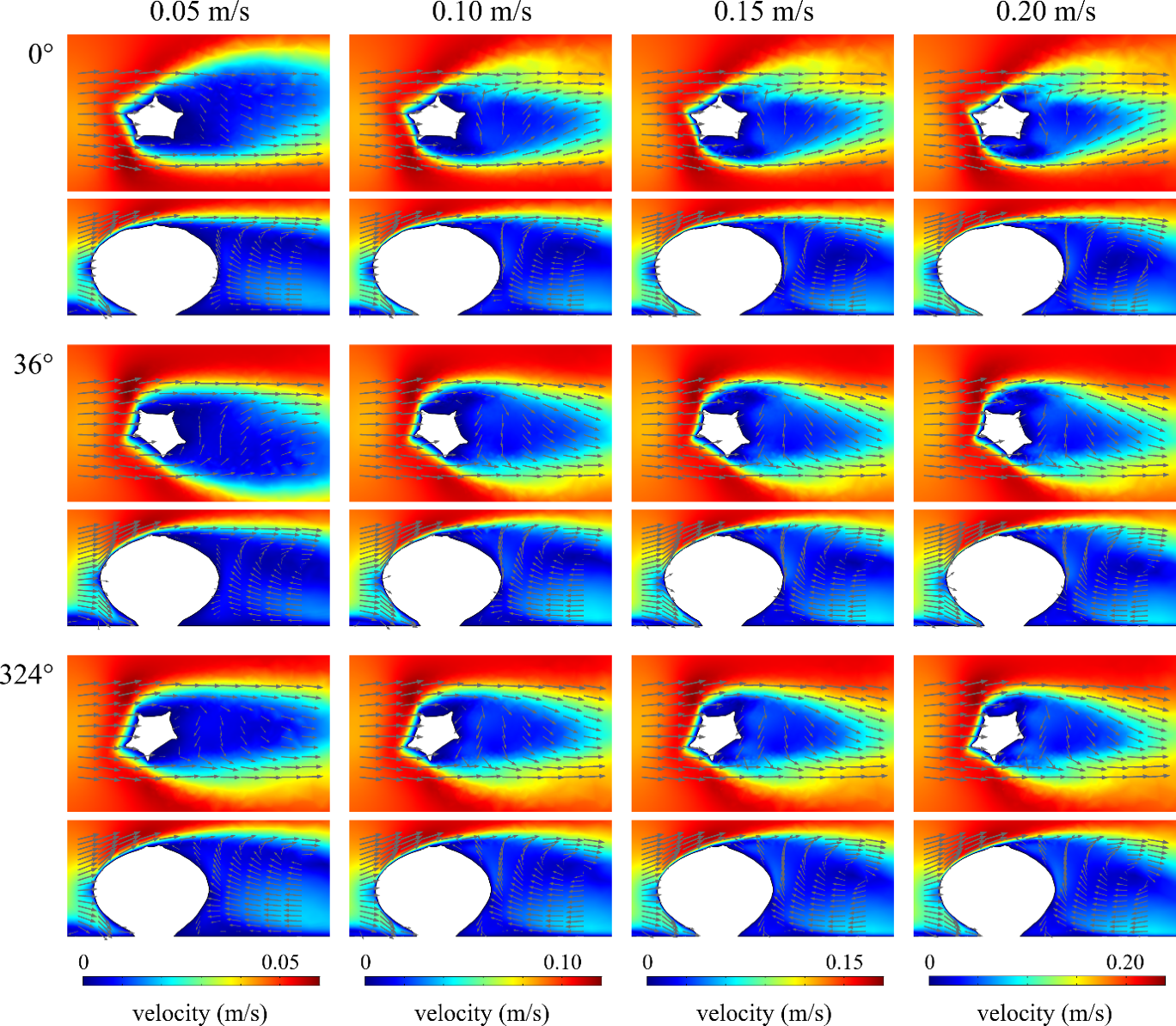
**

**Figure S8.** Two-dimensional surface plots (horizontal and vertical cross-sections) of velocity magnitude with flow vectors (size of arrows proportional to natural logarithm of flow velocity magnitude) at four different inlet velocities (0.05–0.20 m/s) for *Stromatocystites* oriented at 0°, 36° and 324° to the inlet. Direction of ambient flow from left to right.


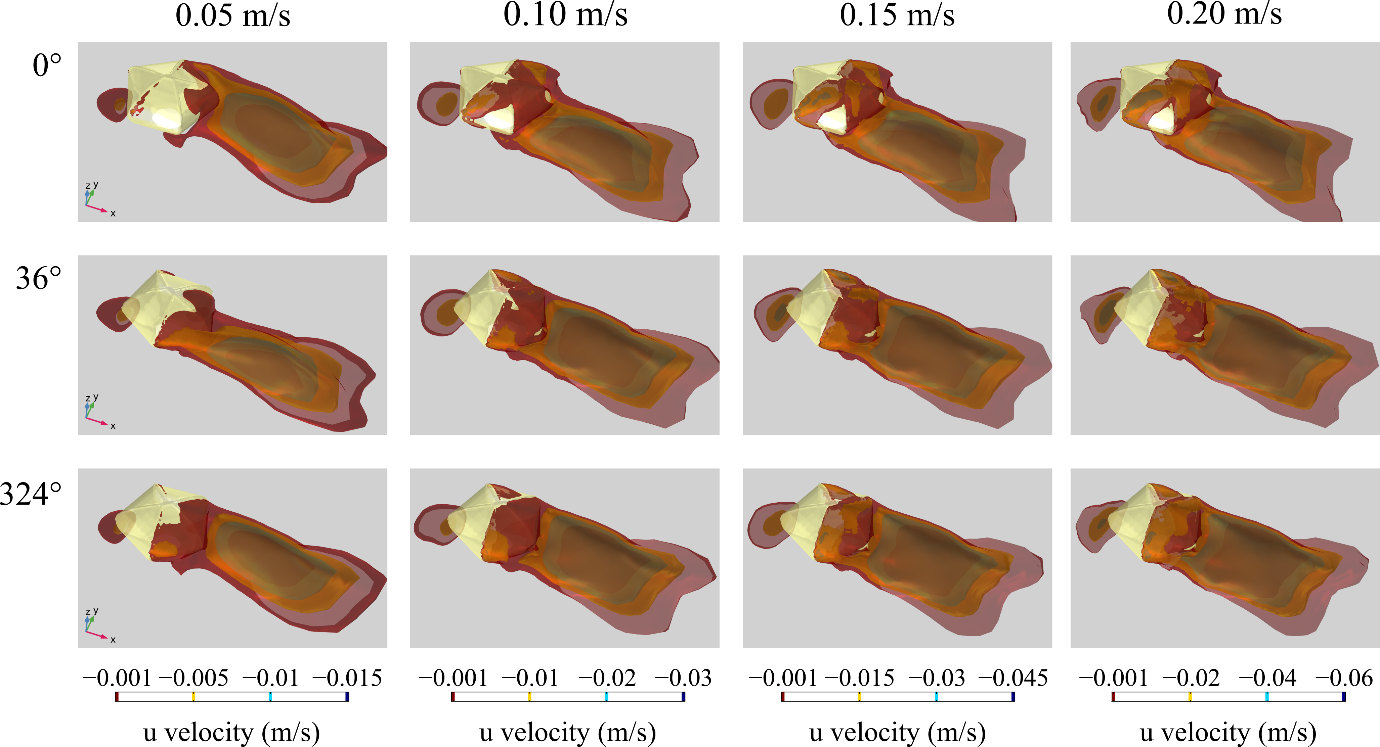


**Figure S9.** Three-dimensional isosurface plots of negative values of velocity component u (parallel to the x-axis) at four different inlet velocities (0.05–0.20 m/s) for *Stromatocystites* oriented at 0°, 36° and 324° to the inlet. Direction of ambient flow from top left to bottom right.


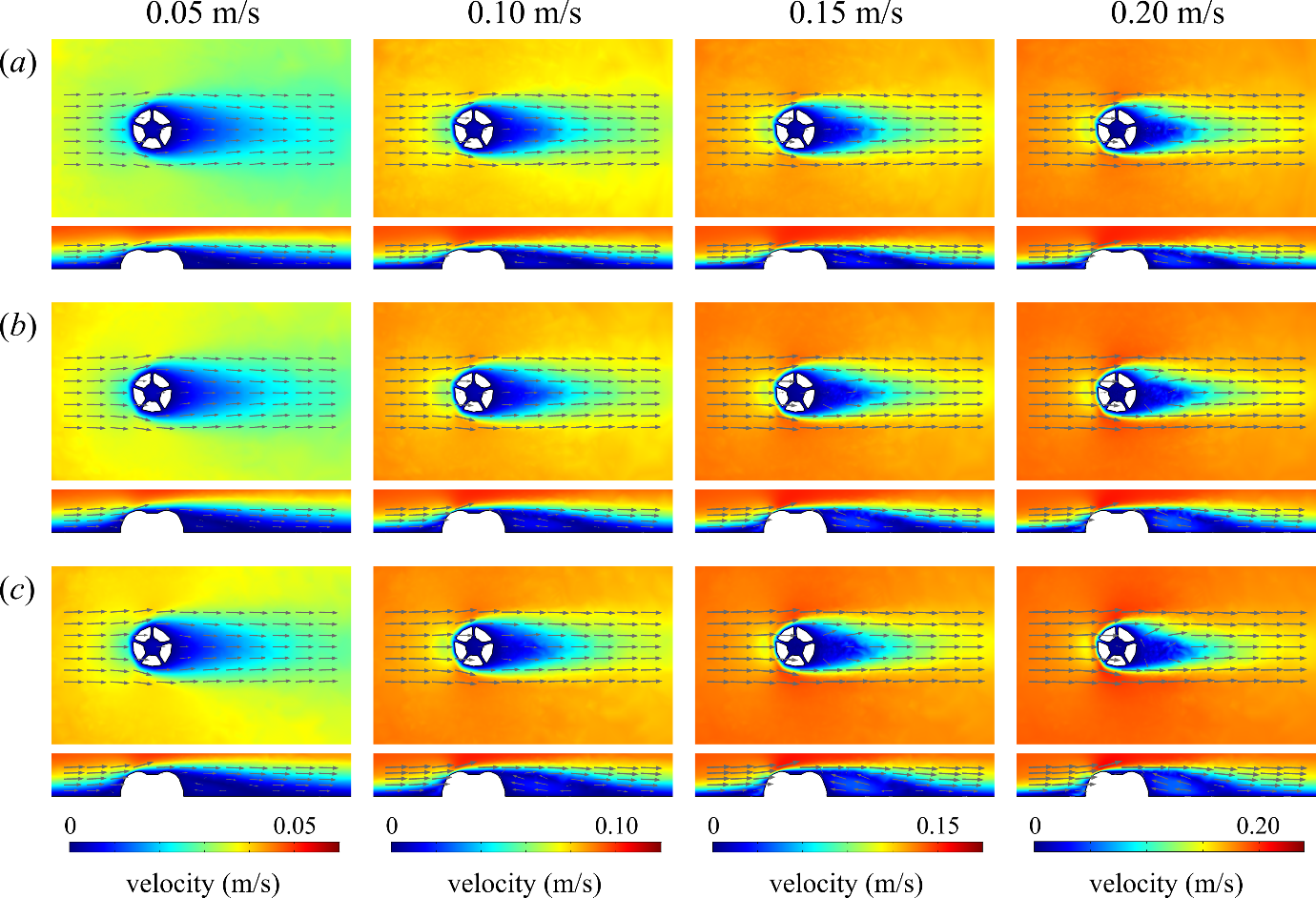


**Figure S10.** Two-dimensional surface plots (horizontal and vertical cross-sections) of velocity magnitude with flow vectors (size of arrows proportional to natural logarithm of flow velocity magnitude) at four different inlet velocities (0.05–0.20 m/s) for *Arkarua* morphotype 1 oriented at 0° to the inlet. (*a*) Original model height. (*b*) Model height increased by 15%. (*c*) Model height increased by 30%. Direction of ambient flow from left to right.


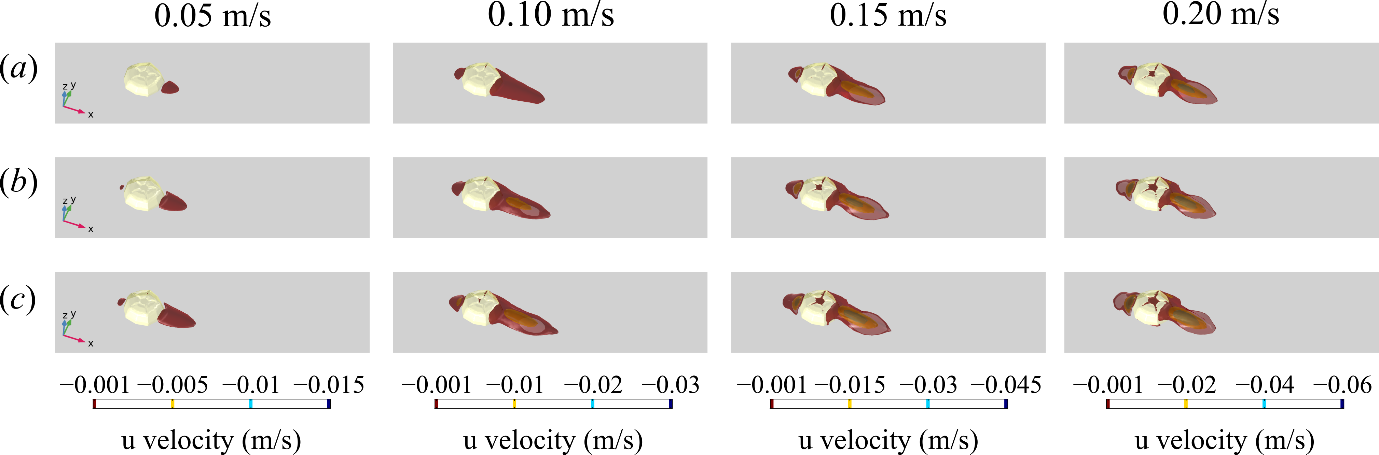


**Figure S11.** Three-dimensional isosurface plots of negative values of velocity component u (parallel to the x-axis) at four different inlet velocities (0.05–0.20 m/s) for *Arkarua* morphotype 1 oriented at 0° to the inlet. (*a*) Original model height. (*b*) Model height increased by 15%. (*c*) Model height increased by 30%. Direction of ambient flow from top left to bottom right.


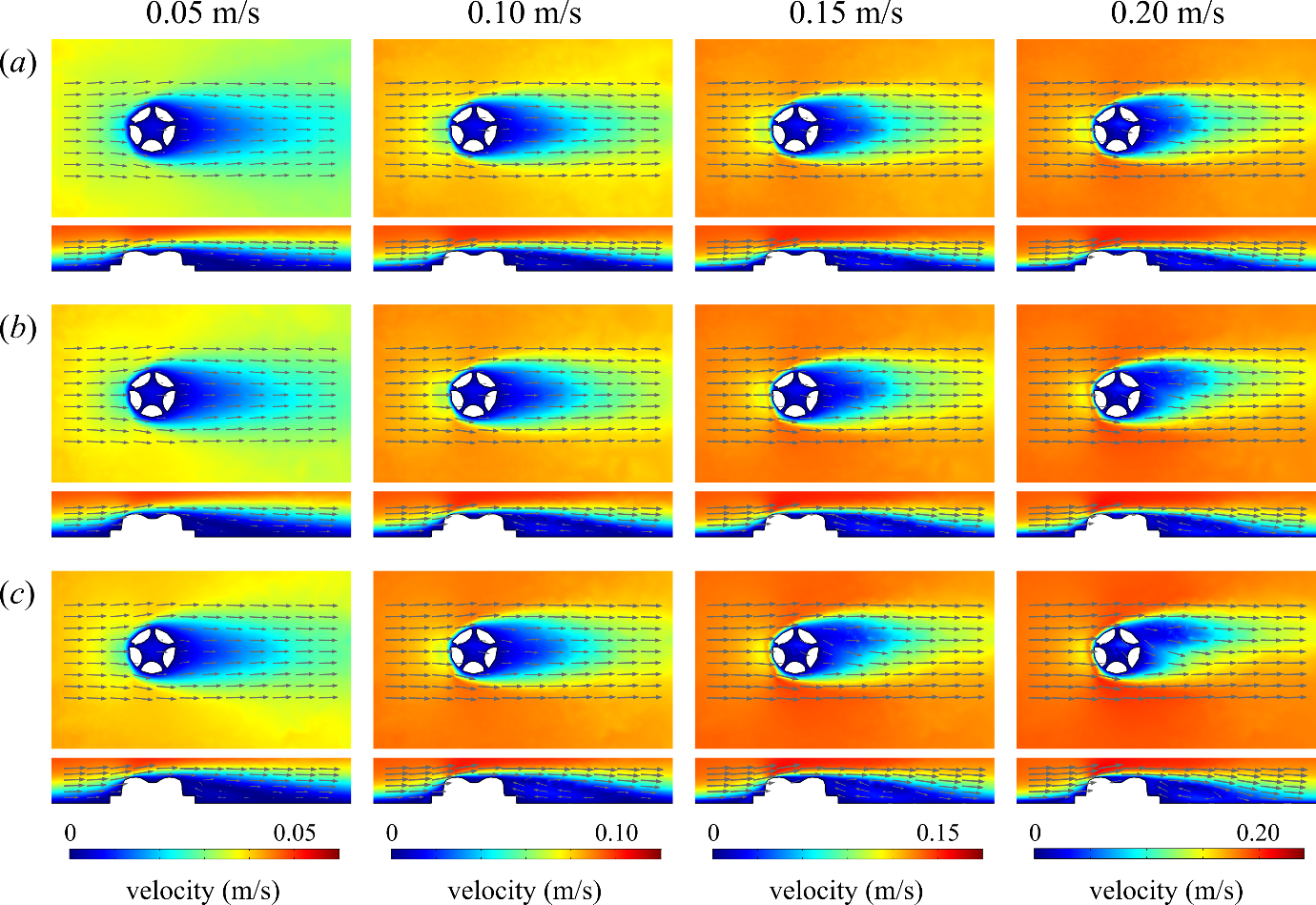


**Figure S12.** Two-dimensional surface plots (horizontal and vertical cross-sections) of velocity magnitude with flow vectors (size of arrows proportional to natural logarithm of flow velocity magnitude) at four different inlet velocities (0.05–0.20 m/s) for *Arkarua* morphotype 2 oriented at 0° to the inlet. (*a*) Original model height. (*b*) Model height increased by 15%. (*c*) Model height increased by 30%. Direction of ambient flow from left to right.


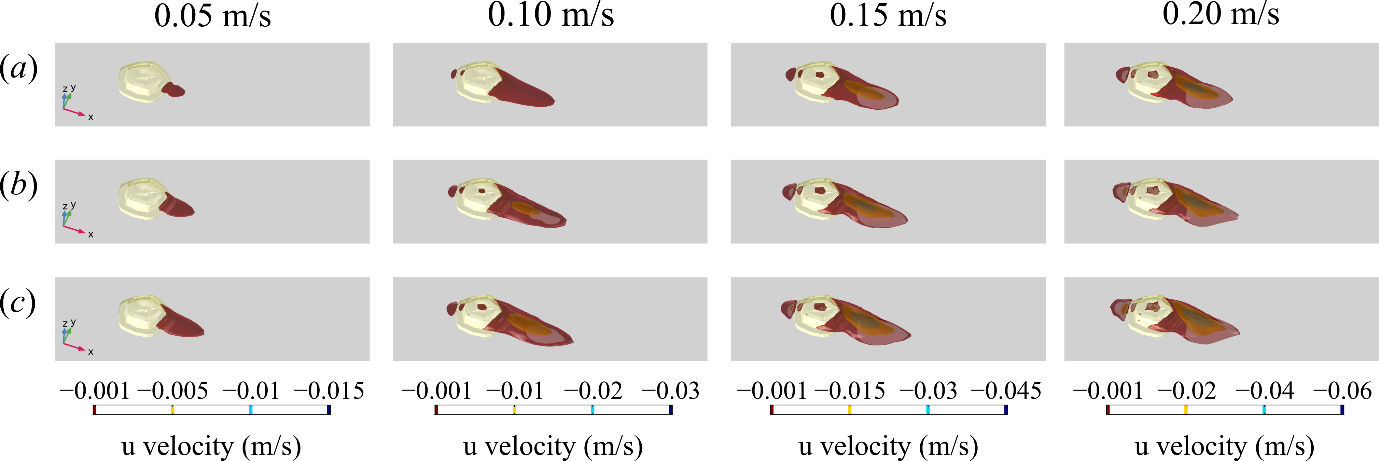


**Figure S13.** Three-dimensional isosurface plots of negative values of velocity component u (parallel to the x-axis) at four different inlet velocities (0.05–0.20 m/s) for *Arkarua* morphotype 2 oriented at 0° to the inlet. (*a*) Original model height. (*b*) Model height increased by 15%. (*c*) Model height increased by 30%. Direction of ambient flow from top left to bottom right.

**Supplementary tables**

**Table S1.** Measurements of *Arkarua* specimens in the collections of the South Australian Museum (SAM).

| **Specimen** | **Maximum internal diameter (mm)** | **Maximum external diameter (mm)** | **Depth (mm)** | **Locality** |
| --- | --- | --- | --- | --- |
| SAM P 26768 | 4.69 | 5.84 | 1.25 | Chace Range, section 31 |
| SAM P 26769 | 5.79 | 10.07 | 1.19 | Chace Range, section 31 |
| SAM P 26770 | 4.47 | 6.22 | 0.87 | Chace Range, section 31 |
| SAM P 26771 | 3.54 | 7.08 | 1.09 | Chace Range, section 31 |
| SAM P 26772 | 3.3 | 6.81 | 0.52 | Chace Range, section 31 |
| SAM P 26773 | 2.26 | 3.85 | 1.3 | Chace Range, section 31 |
| SAM P 26774 | 3.21 | 4 | 1.14 | Chace Range, section 31 |
| SAM P 26775 | 3.56 | 5.19 | 1.66 |  |
| SAM P 40731 | 4.58 | 8.09 | 0.15 |  |
| SAM P 40562 | 4.35 | 7.84 | 1.11 |  |
| SAM P 49266 | 6.16 | 11.68 | 1.11 | Chace Range, section 31 |
| SAM P 26772 B | 2.96 | 5.21 | 0.76 | Chace Range, section 31 |

**Table S2.** Results of sensitivity analysis of mesh size for *Arkarua* morphotype 1.

| **Number of mesh elements** | **Velocity (m/s)** | **Reynolds number** | **Drag force (N)** | **Drag coefficient** | **Difference from coarser mesh** |
| --- | --- | --- | --- | --- | --- |
| 58103 | 0.05 | 290 | 0.00000453 | 0.412 |  |
|  | 0.10 | 580 | 0.00001718 | 0.390 |  |
|  | 0.15 | 870 | 0.00003886 | 0.393 |  |
|  | 0.20 | 1160 | 0.00007047 | 0.400 |  |
|  |  |  |  |  |  |
| 102324 | 0.05 | 290 | 0.00000427 | 0.389 | −5.64% |
|  | 0.10 | 580 | 0.00001618 | 0.368 | −5.80% |
|  | 0.15 | 870 | 0.00003664 | 0.370 | −5.71% |
|  | 0.20 | 1160 | 0.00006639 | 0.377 | −5.78% |
|  |  |  |  |  |  |
| 193751 | 0.05 | 290 | 0.00000408 | 0.371 | −4.50% |
|  | 0.10 | 580 | 0.00001547 | 0.352 | −4.42% |
|  | 0.15 | 870 | 0.00003483 | 0.352 | −4.96% |
|  | 0.20 | 1160 | 0.00006312 | 0.359 | −4.93% |
|  |  |  |  |  |  |
| 372125 | 0.05 | 290 | 0.00000402 | 0.365 | −1.63% |
|  | 0.10 | 580 | 0.00001517 | 0.345 | −1.95% |
|  | 0.15 | 870 | 0.00003387 | 0.342 | −2.74% |
|  | 0.20 | 1160 | 0.00006105 | 0.347 | −3.29% |
|  |  |  |  |  |  |
| 644904 | 0.05 | 290 | 0.00000399 | 0.362 | −0.74% |
|  | 0.10 | 580 | 0.00001495 | 0.340 | −1.41% |
|  | 0.15 | 870 | 0.00003311 | 0.334 | −2.26% |
|  | 0.20 | 1160 | 0.00005932 | 0.337 | −2.83% |

**Table S3.** Results of sensitivity analysis of mesh size for *Arkarua* morphotype 2.

| **Number of mesh elements** | **Velocity (m/s)** | **Reynolds number** | **Drag force (N)** | **Drag coefficient** | **Difference from coarser mesh** |
| --- | --- | --- | --- | --- | --- |
| 80442 | 0.05 | 370 | 0.00000546 | 0.420 |  |
|  | 0.10 | 740 | 0.00002044 | 0.393 |  |
|  | 0.15 | 1110 | 0.00004564 | 0.390 |  |
|  | 0.20 | 1480 | 0.00008155 | 0.392 |  |
|  |  |  |  |  |  |
| 137099 | 0.05 | 370 | 0.00000521 | 0.401 | −4.51% |
|  | 0.10 | 740 | 0.00001948 | 0.375 | −4.69% |
|  | 0.15 | 1110 | 0.00004362 | 0.373 | −4.43% |
|  | 0.20 | 1480 | 0.00007839 | 0.377 | −3.87% |
|  |  |  |  |  |  |
| 281648 | 0.05 | 370 | 0.00000509 | 0.391 | −2.42% |
|  | 0.10 | 740 | 0.00001897 | 0.365 | −2.61% |
|  | 0.15 | 1110 | 0.00004218 | 0.360 | −3.30% |
|  | 0.20 | 1480 | 0.00007562 | 0.364 | −3.54% |
|  |  |  |  |  |  |
| 530589 | 0.05 | 370 | 0.00000499 | 0.384 | −1.97% |
|  | 0.10 | 740 | 0.00001833 | 0.353 | −3.35% |
|  | 0.15 | 1110 | 0.00004052 | 0.346 | −3.92% |
|  | 0.20 | 1480 | 0.00007274 | 0.350 | −3.81% |
|  |  |  |  |  |  |
| 828418 | 0.05 | 370 | 0.00000486 | 0.374 | −2.49% |
|  | 0.10 | 740 | 0.00001786 | 0.343 | −2.58% |
|  | 0.15 | 1110 | 0.00003938 | 0.337 | −2.81% |
|  | 0.20 | 1480 | 0.00007072 | 0.340 | −2.77% |

**Table S4.** Results of sensitivity analysis of mesh size for *Cambraster*.

| **Number of mesh elements** | **Velocity (m/s)** | **Reynolds number** | **Drag force (N)** | **Drag coefficient** | **Difference from coarser mesh** |
| --- | --- | --- | --- | --- | --- |
| 86682 | 0.05 | 610 | 0.00001806 | 0.463 |  |
|  | 0.10 | 1220 | 0.00007075 | 0.454 |  |
|  | 0.15 | 1830 | 0.00016330 | 0.465 |  |
|  | 0.20 | 2440 | 0.00029742 | 0.477 |  |
|  |  |  |  |  |  |
| 147833 | 0.05 | 610 | 0.00001738 | 0.446 | −3.76% |
|  | 0.10 | 1220 | 0.00006864 | 0.440 | −2.98% |
|  | 0.15 | 1830 | 0.00015833 | 0.451 | −3.05% |
|  | 0.20 | 2440 | 0.00028627 | 0.459 | −3.75% |
|  |  |  |  |  |  |
| 289166 | 0.05 | 610 | 0.00001674 | 0.429 | −3.68% |
|  | 0.10 | 1220 | 0.00006654 | 0.427 | −3.07% |
|  | 0.15 | 1830 | 0.00015542 | 0.443 | −1.83% |
|  | 0.20 | 2440 | 0.00028628 | 0.459 | 0.01% |
|  |  |  |  |  |  |
| 555026 | 0.05 | 610 | 0.00001630 | 0.418 | −2.65% |
|  | 0.10 | 1220 | 0.00006430 | 0.412 | −3.37% |
|  | 0.15 | 1830 | 0.00015011 | 0.428 | −3.42% |
|  | 0.20 | 2440 | 0.00027546 | 0.441 | −3.78% |
|  |  |  |  |  |  |
| 1017980 | 0.05 | 610 | 0.00001605 | 0.412 | −1.52% |
|  | 0.10 | 1220 | 0.00006300 | 0.404 | −2.02% |
|  | 0.15 | 1830 | 0.00014694 | 0.419 | −2.11% |
|  | 0.20 | 2440 | 0.00026833 | 0.430 | −2.59% |

**Table S5.** Results of sensitivity analysis of mesh size for *Stromatocystites*.

| **Number of mesh elements** | **Velocity (m/s)** | **Reynolds number** | **Drag force (N)** | **Drag coefficient** | **Difference from coarser mesh** |
| --- | --- | --- | --- | --- | --- |
| 72801 | 0.05 | 635 | 0.00008613 | 0.834 |  |
|  | 0.10 | 1270 | 0.00033883 | 0.820 |  |
|  | 0.15 | 1905 | 0.00075827 | 0.816 |  |
|  | 0.20 | 2540 | 0.00134331 | 0.813 |  |
|  |  |  |  |  |  |
| 123087 | 0.05 | 635 | 0.00008126 | 0.787 | −5.65% |
|  | 0.10 | 1270 | 0.00031527 | 0.763 | −6.95% |
|  | 0.15 | 1905 | 0.00070271 | 0.756 | −7.33% |
|  | 0.20 | 2540 | 0.00124512 | 0.754 | −7.31% |
|  |  |  |  |  |  |
| 223797 | 0.05 | 635 | 0.00007930 | 0.768 | −2.41% |
|  | 0.10 | 1270 | 0.00030750 | 0.745 | −2.47% |
|  | 0.15 | 1905 | 0.00068166 | 0.734 | −3.00% |
|  | 0.20 | 2540 | 0.00120214 | 0.728 | −3.45% |
|  |  |  |  |  |  |
| 432389 | 0.05 | 635 | 0.00007710 | 0.747 | −2.77% |
|  | 0.10 | 1270 | 0.00029395 | 0.712 | −4.41% |
|  | 0.15 | 1905 | 0.00065783 | 0.708 | −3.49% |
|  | 0.20 | 2540 | 0.00116273 | 0.704 | −3.28% |
|  |  |  |  |  |  |
| 845973 | 0.05 | 635 | 0.00007629 | 0.739 | −1.06% |
|  | 0.10 | 1270 | 0.00029001 | 0.702 | −1.34% |
|  | 0.15 | 1905 | 0.00065017 | 0.700 | −1.16% |
|  | 0.20 | 2540 | 0.00115190 | 0.697 | −0.93% |
